# One-pot fabrication of Ag @Ag_2_O core–shell nanostructures for biosafe antimicrobial and antibiofilm applications

**DOI:** 10.1038/s41598-021-01687-4

**Published:** 2021-11-19

**Authors:** Shaimaa Elyamny, Marwa Eltarahony, Marwa Abu-Serie, Marwa M. Nabil, Abd El-Hady B. Kashyout

**Affiliations:** 1 Electronic Materials Research Department, Advanced Technology and New Materials Research Institute, City of Scientific Research and Technological Applications (SRTA-City), P.O. Box 21934, New Borg El-Arab City, Alexandria Egypt; 2 Environmental Biotechnology Department, Genetic Engineering and Biotechnology Research Institute (GEBRI), City of Scientific Research and Technological Applications (SRTA-City), New Borg El-Arab City, Alexandria 21934 Egypt; 3 Medical Biotechnology Department, Genetic Engineering and Biotechnology Research Institute, (GEBRI), City of Scientific Research and Technological Applications (SRTA-City), New Borg El-Arab City, Alexandria 21934 Egypt

**Keywords:** Biological techniques, Biotechnology, Microbiology, Medical research, Chemistry, Materials science, Nanoscience and technology, Physics

## Abstract

Microbial contamination is one of the major dreadful problems that raises hospitalization, morbidity and mortality rates globally, which subsequently obstructs socio-economic progress. The continuous misuse and overutilization of antibiotics participate mainly in the emergence of microbial resistance. To circumvent such a multidrug-resistance phenomenon, well-defined nanocomposite structures have recently been employed. In the current study, a facile, novel and cost-effective approach was applied to synthesize Ag@Ag_2_O core–shell nanocomposites (NCs) via chemical method. Several techniques were used to determine the structural, morphological, and optical characteristics of the as-prepared NCs. XRD, Raman, FTIR, XPS and SAED analysis revealed a crystalline hybrid structure of Ag core and Ag_2_O shell. Besides, SEM and HRTEM micrographs depicted spherical nanoparticles with size range of 19–60 nm. Additionally, zeta potential and fluorescence spectra illustrated aggregated nature of Ag@Ag_2_O NCs by − 5.34 mV with fluorescence emission peak at 498 nm. Ag@Ag_2_O NCs exhibited higher antimicrobial, antibiofilm, and algicidal activity in dose-dependent behavior. Interestingly, a remarkable mycocidal potency by 50 μg of Ag@Ag_2_O NCs against *Candida albican*; implying promising activity against COVID-19 white fungal post-infections. Through assessing cytotoxicity, Ag@Ag_2_O NCs exhibited higher safety against Vero cells than bulk silver nitrate by more than 100-fold.

## Introduction

Earth’s biosphere is occupied by a plethora of microorganisms which encompass several categories, including bacteria, archaea, yeast, molds, algae, viruses and protozoa. Several benefits are provided by them in maintaining a balanced ecosystem, such as oxygen generation, nutrient supplementation, organic material decomposing and bioactive compounds production. Nonetheless, their pathogenicity represents a serious problem for public health and the entire ecosystem. The microbes causing infectious diseases are ubiquitous through several routes such as food manufacturing machines, water purification systems and polluted medical devices^1^. As a result, various antimicrobial agents, particularly antibiotics, were developed to combat the spread of pathogen-causing infections. However, the intense and widespread abuse of such biocides led to emergence of multi-drug resistant microbes (MDR). Recently, nanotechnology with its related products opens different avenues to face and solve the MDR threat. The metal and metal oxide nanoparticles either sole or in nanocomposite structures increased the antimicrobial activity by the virtue of expanding spectrum of enhanced features^2^.

Silver nanoparticles (Ag Nps), are among the most attractive nanomaterials have been widely used in a range of biomedical applications, including diagnosis treatment, drug delivery, medical device coating, and for personal health care^3^. For years, knowledge about silver’s ability to kill harmful bacteria has made its nanoparticles popular for creating various products. Silver has many advantages, including the fact that it is non-toxic to humans at very low doses and the use of silver was a common expedient for cooking procedures and for preserving water from contamination. Previous studies have presented improved bactericidal activity for lower nanoparticle sizes associated with higher surface area of the nanomaterials^4^. Silver ions are known to specifically react with the metabolic enzymes inside the bacteria; causing growth suppression. An oxide shell has also been demonstrated to boost biocidal action^5^. The preparation process of good quality nanoparticles (NPs) is vital to ensure their multi-directional effectiveness^6^. Several chemical and physical means were employed to synthesize NPs, the physical one involving laser ablation of a solid target in water^7^, condensation or evaporation and the thermal treatment of Ag NPs in an organic solvent at temperatures up to 360 °C in the gas atmosphere^8^. In the laser ablation procedure, the lack of any chemical reagents provides a unique benefit, but it is an expensive method. Chemical methods are alternatively applied, in which metal nanoparticles sizes are reduced leading to the formation of minute metal clusters. The synthesis of the nanoparticles in solution has important advantages as the ease with which the design, shape and size of the nanoparticles can be precisely controlled^9^.

In the present study, Ag@Ag_2_O core–shell nanocomposites (NCs) were synthesized via a simple chemical method. The as-prepared NCs were characterized structurally, morphologically and optically. Thereafter, the antimicrobial and antibiofilm efficiency of Ag@Ag_2_O nanocomposite against planktonic and biofilm-forming pathogens were evaluated. Additionally, the biocompatibility of Ag@Ag_2_O nanocomposites was assessed.

## Materials and methods

### Methodology

**Figure 1:**
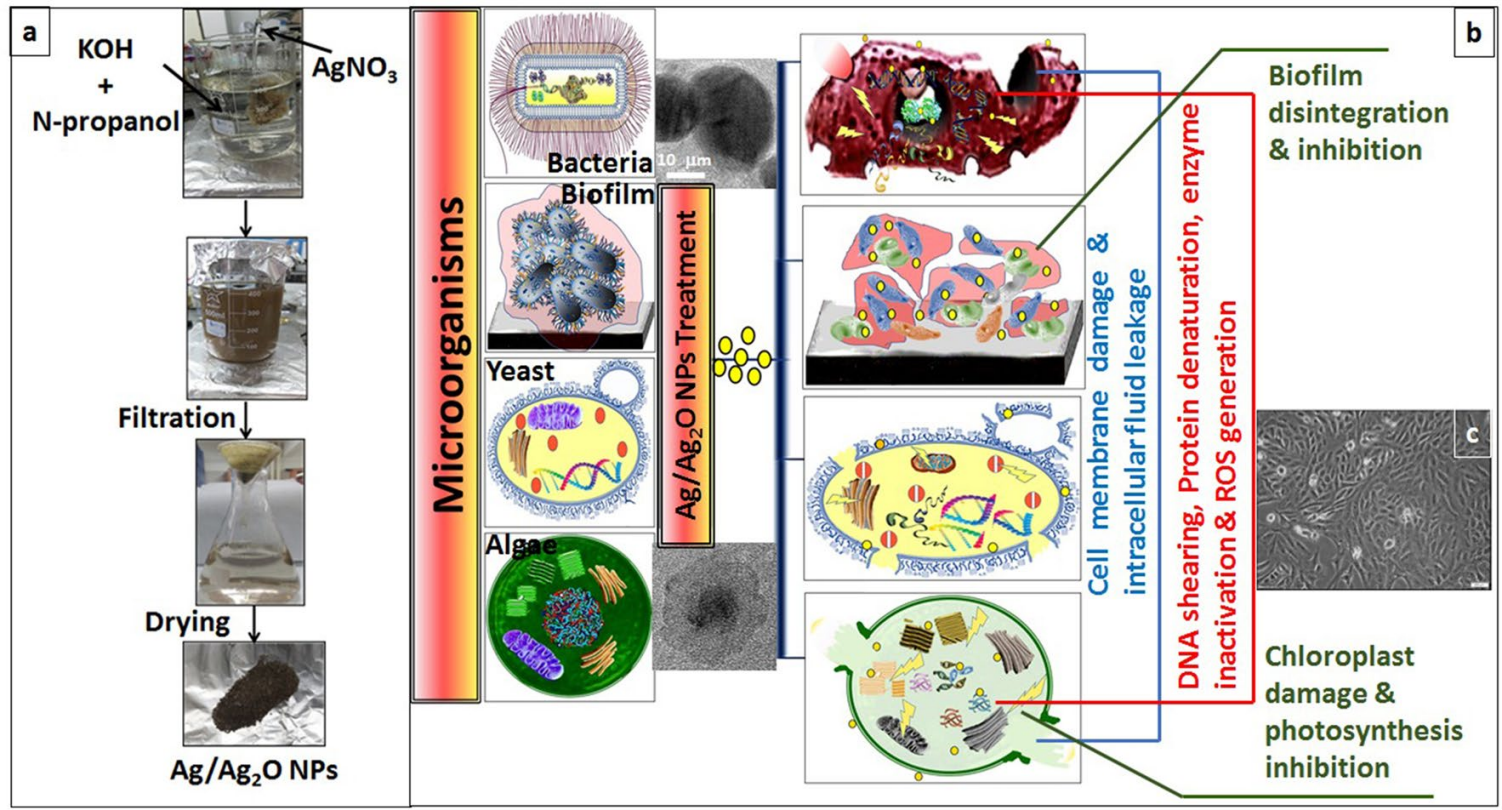
Schematic diagram of (**a**) synthesis of Ag@Ag_2_O NCs, (**b**) Antimicrobial activity and (**c**) Cytotoxicity.

The formation process of nano-sized silver composites (Ag@Ag_2_O) powder is a simple and safe system using alkali chemical techniques. 0.1 N of silver nitrate (AgNO_3_, 99.97%, Sigma Aldrich) aqueous solution which is used as a precursor of the silver element. It's added drop-wise to an alkali mixture solution, which contains [2.5 Wt% of Potassium Hydroxide (KOH, 99.97%, Sigma Aldrich), 10 Vol% of n-propanol (NPA) and deionized water, with stirring]. The reaction temperature is kept constant at 70–80 °C for 2 h. The solution was constantly stirred with a magnetic stir bar, until the solution turned into a grey colloidal suspension; indicating the fulfillment of the chemical reaction. Then, the precipitate powder is filtered, and dried at 60 °C overnight, as shown in Fig. 1a.

### Characterization methods

The structural, composition and morphological properties of the (Ag@Ag_2_O) NCs composite powder were investigated using X-ray diffraction (XRD), Raman spectroscopy, Fourier-transform infrared spectroscopy (FTIR), scanning electron microscopy (SEM), high resolution transmission electron microscopy (HRTEM), zeta potential and Fluorescence spectra. X-ray diffraction measurement was performed using Shimadzu 7000 XRD, with CuKα radiation (*λ* = 1.54 Å) generated at 30 mA and 30 kV with a scanning rate of 4° min^−1^ and 2θ values ranged between 25° and 80°. Raman spectrum was obtained at an excitation wavelength of 532 nm using Raman spectroscopy (Senterra, Germany). For the determination of the chemical bonds formed during the preparation process, Fourier Transform Infrared Spectrophotometer (FTIR, Bruker Corporation, Ettlingen, Germany) is used. The powder product morphology was investigated using Scanning Electron Microscopy [SEM, JEOL (JSM 5300)]. However, high resolution transmission electron microscope TEM (HR-TEM, JEOL-2100, Japan) was employed to examine morphology, high resolution d-spacing of the different structures, electron diffraction and mapping of silver and oxygen elements. X-Ray photospectroscopy (XPS) measurement was carried out using PHI 5000 Versa Probe III Scanning XPS Microprobe with Monochromatic Al source ranged from 0–1486.6 eV. Electrostatic potential was determined by the DLS technique using Zetasizer Nano ZS (Malvern Instruments, Worcestershire, UK) and the data were analyzed by Zetasizer software 6. Finally, the fluorescence spectrum was recorded at an excitation wavelength of 250 nm on fluorescence spectrophotometer (Agilent, G9800A, USA).

### Determination of antimicrobial efficiency of Ag@Ag_2_O NCs against planktonic pathogens

The antibacterial and antifungal activity of the as-synthesized Ag@Ag_2_ONCs in comparison to AgNO_3_ (Silver precursor) was screened against various Gram-negative [*Escherichia coli* (ATCC − 25,922)*, Pseudomonas aeruginosa* (ATCC − 27,853), *Salmonellatyphi* (ATTC − 700,931)], Gram-positive bacteria [*Staphylococcus aureus* (ATCC − 25,923), *Bacillus cereus* (ATCC − 7464), *Enterococcus faecalis* (ATCC − 29,212)] and yeast *Candidaalbicans* (ATCC − 10,231) via well diffusion assay. The sterile agar plate sets (Müller-Hinton agar for bacteria and yeast extract peptone dextrose agar (YPD) for yeast) were swabbed homogeneously using sterile cotton-tipped applicators with 100 μL of 10^6^ CFU/mL of each examined organism. A sterile cork-borer (5 mm) was used to make the wells. Different concentrations (5, 10, 25, 50 and100 µg/mL) of Ag@/Ag_2_ONCs and AgNO_3_ were loaded separately in each well. The bacterially inoculated plate sets were incubated at 37 °C for 24 h. For yeast, the plates were incubated at 25 °C ± 2 °C for 2–3 days. The plates were examined after incubation for the presence of a zone of inhibition (ZOI), which were measured and expressed in millimetres (mm). It was calculated by subtracting the well diameter from the total inhibition zone diameter^10^.

### Evaluation of the as-prepared Ag@Ag_2_ONCs as anti-bio-film agent

The inhibitory effect of Ag@Ag_2_ONCs and AgNO_3_ (5, 10, 25, 50 and100 µg/mL) against *P. aeruginosa* and *S. aureus* biofilms were assessed using tissue culture plate method. A sterile polystyrene 96-well microplate was seeded by 100 μL of tryptone soy broth (TSB) containing 10^8^ CFU/mL of each tested strain. Simultaneously, two controls were run in parallel; positive control wells (medium containing a bacterial culture) and negative control wells (sterile TSB only). After 24 h of static incubation at 37 °C, washing, fixation and staining of the remained biofilm were carried out by 95% ethanol and 0.25% crystal violet, respectively. The absorbencies of adhered cells were measured spectrophotometrically at 595 nm. All the experiments were carried out in triplicate and the results are expressed as mean ± SD^11^. The following equation was employed to calculate inhibition percentage of biofilm formation

1 $$Inhibition~percentage~of~adhesion=\frac{A-{A}_{o}}{A}\times 100$$

where A represents the absorbance of the positive control wells and A_0_ reveals the absorbance of the treated wells containing an antimicrobial agent.

### Biofilm disintegrating assay

The potential of Ag@Ag_2_ONCs to degrade the already formed biofilms by *P. aeruginosa* and *S. aureus* were examined in comparison to AgNO_3_. Firstly, the bacterial lawn (10^8^ CFU/mL) was inoculated into 96-well microplates and incubated statically at 37 °C for 24 h to permit biofilm formation. Secondly, the well contents were discarded aseptically. The diluted Ag@Ag_2_ONCs and AgNO_3_ to concentrations (5, 10, 25, 50 and100 µg/mL) were added to each well. The incubation, processing, quantification and disintegration percentage of the biofilms were performed as previously described. All the experiments were carried out in triplicate and the results are expressed as mean ± SD. As stated by Cremonini et al.^12^ the biofilm was deemed strong, medium and low at optical density (OD) ˃ 2, 1 ˂ OD ˂ 2 and 0.5 ˂ OD ˂1, respectively.

### Antagonistic effect of Ag@/Ag_2_O NCs on the algal growth

The inhibitory effect of Ag@Ag_2_O NCs was evaluated against *Chlorella vulgaris* by adding (5, 10, 25, 50 and100 µg/mL) in parallel to exact concentrations of AgNO_3_. The algae were propagated in sterilized Bold’s basal media (BBM) medium; incubated at 25 °C under illumination with daily cycles of 12-h light and 12-h night for 7 days^13^. The cell count was assessed with a hemocytometer under a light microscope (Olympus BH-2, Japan). The inhibition percentage was calculated as mentioned in Eq. (1), and the results are expressed as means ± SD.

### Investigation of the cytotoxicity of Ag@Ag_2_ONCs comparing with silver nitrate against normal cells

Normal mammalian kidney epithelial cells (Vero) were used to detect cytotoxicity of the studied compounds. Vero cell line was cultured in DMEM medium-contained 10% fetal bovine serum (FBS), seeded as 4 × 10^3^ cells per well in 96-well cell culture plate and incubated at 37ºC in 5% CO_2_ incubator. After 24 h for cell attachment, serial concentrations of Ag@Ag_2_O NCsand silver nitrate were incubated with Vero cells for 72 h. Cell viability was assayed by MTT method^14^. Twenty microliters of 5 mg/mL MTT (Sigma, USA) was added to each well and the plate was incubated at 37 °C for 3 h. After removing the MTT solution, 100 µL DMSO was added and the absorbance of each well was measured at 570 nm using a microplate reader (BMG LabTech, Germany). The effective safe concentration (EC_100_) value (at 100% cell viability) of the tested compounds was estimated by the Graphpad Instat software.

## Results and discussions

### Structural analysis and chemical bonds formation

#### X-ray diffraction (XRD)

**Figure 2:**
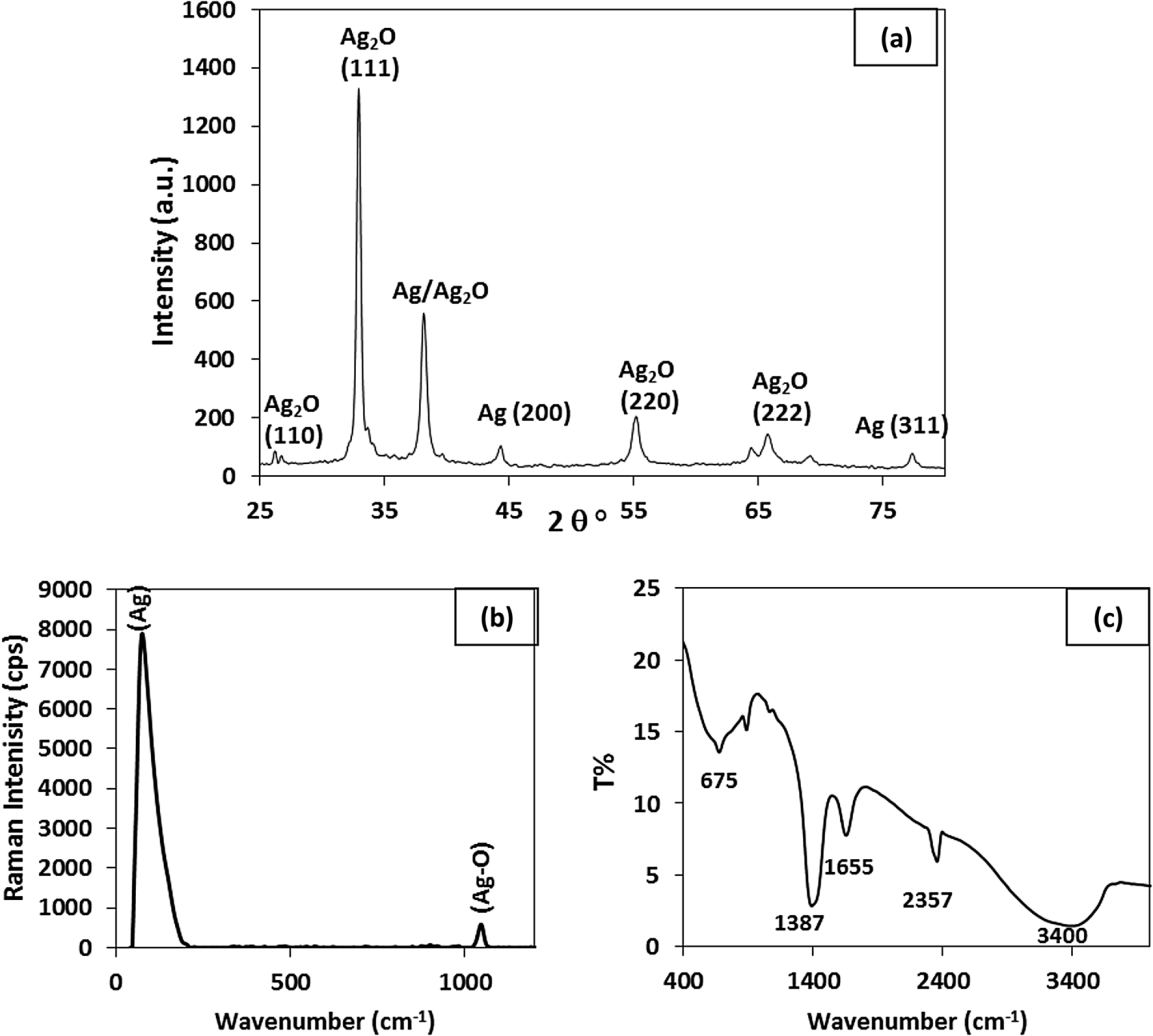
(**a**) XRD pattern, (**b**) Raman Spectrum and (**c**) FTIR spectrum of the Ag@Ag_2_O NCs.

The X-ray diffraction (XRD) spectrum of nano-composite (Ag@Ag_2_O)NCsis given in Fig. 2a. The peaks observed at the diffraction angles (2θ): 26.2°, 32.9°, 38.1°, 55.2°, 65.7° and 69.1° corresponding to (110), (111), (200), (220), (311) and (222) set of Ag_2_O lattice planes (cubic structure), respectively [JCPDS card No. 76-1393]. The high intense peak (111) may refer to the arrangement of lattice atoms in an ordered structural fashion^15^. Whiles, the peaks observed at (2θ): 44.34° and 77.34° correspond to the metallic Ag (200) and (311), respectively in the face-centered cubic structure [JCPDS card No. 04-0783]^16^.On the other hand, the close overlap between Ag and Ag_2_O diffraction peaks and the difficulty to distinguish between the Ag^+^ and Ag^0^ peaks at the diffraction angle of 38.1° inferred a formation of a hybrid structure^17,18^. Sajjad Ullah et al.^18^ found that, the diffraction peak at around 38° could be assigned to metallic Ag and/or Ag_2_O and proving the existence of Ag (38.1°, JCPDS card No. 65-2871 and 04-0783) and Ag_2_O at the same time (38.0°, JCPDS card No. 41-1104) in the samples. The structure could possibly have an Ag_2_O shell with Ag as the core with a decreasing gradient of oxygen from the surface to the core^6^. Despite the simplicity of preparation method, the silver element needs a special medium during its preparation. The individual crystallite size (t) was calculated using Scherrer’s formula^19^ given by Eq. (2).

2 $$t= k.\lambda / \beta .\cos \theta$$

where *k* is the Scherrer’s constant (0.89–0.9), *λ* is the X-ray wavelength, *β* is the full width at half maximum (FWHM) and *θ* is the Bragg angle^19^. According to Eq. (2), the sample crystallite size for the plan (111) is calculated and is found to be approximately 18.6 nm.

#### Raman analysis

The molecular structure and phase identification of the Ag@Ag_2_Ocore shell are explored using Raman spectra. Figure 2b shows the Raman spectrum for the prepared nanopowder in a range from 0 to 4500 cm^−1^. Two major peaks are clearly detected; the first one at 74.1 cm^−1^ with an intensity of 7900 cps for Ag^0^ (Ag lattice vibrational mode) and the other one at 1046.7 cm^−1^ with an intensity of 583 cps for Ag_2_O (Ag–O stretching/bending modes)^20^.

#### Fourier-transform infrared spectroscopy (FTIR):

FTIR analysis reveals the functional groups of the Ag@/Ag_2_O nanocomposite synthesized using alkali chemical treatment (Fig. 2c).The broad band at3400 cm^-1^indicates the O–H stretching vibrations of the hydroxyl groups^21^ corresponding to H-bonded alcohols and also to intramolecular H bonds which are most probably from water molecules^22^. The peaks at 2357 cm^−1^and 1655 cm^−1^prove the existence of O–H carboxylic acids^23^ and OH bending, respectively^24^. The band at 1387 cm^−1^ assigned to O–H bend of carboxylate^25^. The absorption band on 675 cm ^−1^ is due to Ag–O stretching mode, which corresponds to Ag–O vibration in Ag_2_O.Furthermore, the appearance of follower peak at 868.68 cm^−1^corresponds to the metal–oxygen vibrations for the formation of (Ag@Ag_2_O) NCs^15^; thus, synchronizing with the aforementioned XRD results; confirming the formation of (Ag@Ag_2_O) NCs.

#### X-ray photoelectron spectroscopy (XPS)

**Figure 3:**
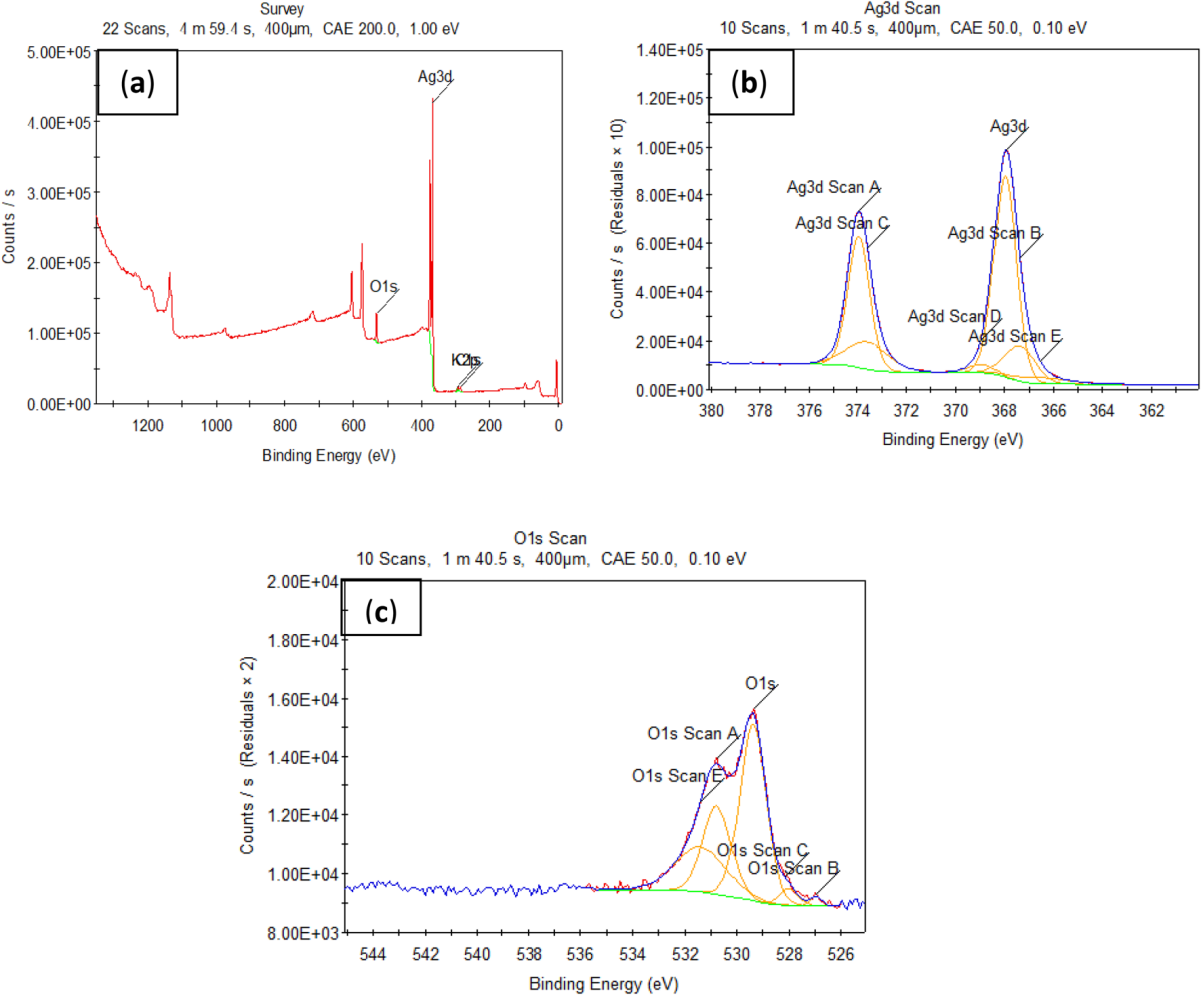
XPS analyses of the Ag@Ag_2_O NCs; (**a**) Survey, (**b**) Ag3*d* and (**c**) O1*s*.

The XPS data for the chemically prepared Ag@Ag_2_O NCs is illustrated in Fig. 3. Figure 3a shows the general survey analysis of the nanopowder, which exhibits a major detected peak of the Ag3d at a binding energy of 368.34 eV with an atomic ratio of 48.5%. Also, O1s peak is detected at a binding energy of 530.81 eV with an atomic ratio of 29.75% and K2p peak is observed at a binding energy of 293.5 eV with an atomic ratio of 2.18%. Finally, C1s peak is measured at a binding energy of 285.21 eV with an atomic ratio of 19.56%. In Fig. 3b, the high resolution of the Ag3d spectrum displays two main strong bands. Such two bands can be further de-convoluted into two pairs of sub-peaks. The peaks at 367.98 eV with an atomic ratio of 44.01% and 373.96 eV with an atomic ratio of 28.75% are respectively assigned to Ag^0^ (3*d*5/2 and 3*d*3/2). The other set of bands is detected at 367.38 eV with an atomic ratio of 10.48% and 373.6 eV with an atomic ratio of 10.36% are attributed to Ag^+^ (3*d*5/2 and 3*d*3/2, respectively) in the nanocomposite. Figure 3c confirms the oxidation of the silver nanoparticles through the existence of the O1*s* spectrum at 529.39 eV with an atomic ratio of 47.64%, at 530.77 eV with an atomic ratio of 25.32% and at 531.4 eV with an atomic ratio of 23.19%. Finally, the results confirm that there are two different configurations of silver species, namely Ag_2_O and Ag, which is consistent with many published reports^26–28^. The detected elemental carbon in the main survey analysis may have originated from the ambient atmosphere itself. The adsorption of hydrocarbons from the surrounding atmosphere, which results in the creation of a thin carbon layer on surfaces, is most likely the source of the carbon contamination^29^.

### Morphological analysis

#### Scanning electron microscope (SEM)

**Figure 4:**
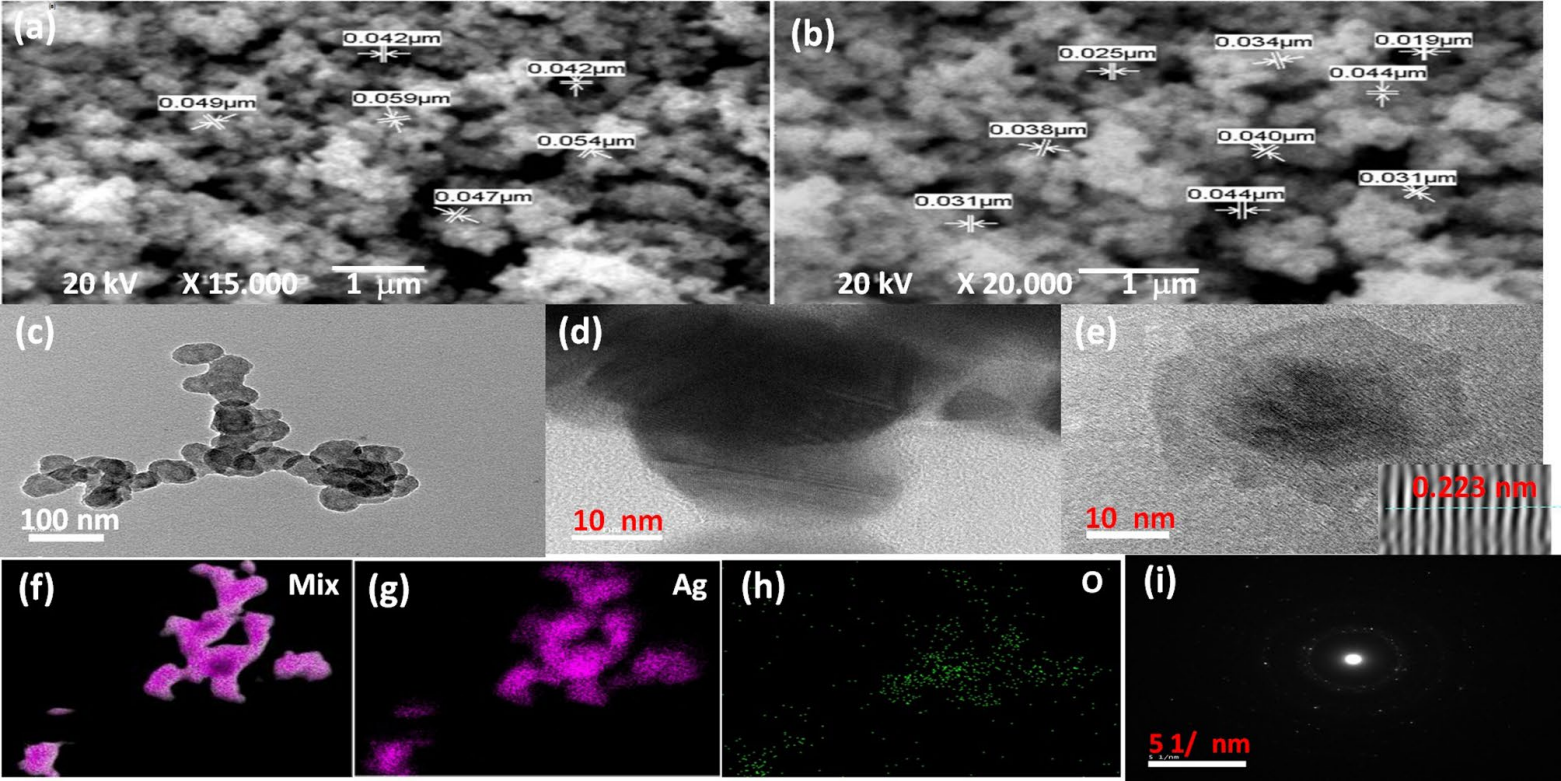
(**a**, **b**) SEM images at different magnifications, (**c**) TEM, (**d**, **e**) HRTEM, (**f**–**h**) elemental mapping distribution and (**i**) SAED pattern of the Ag@Ag_2_O NCs.

Figure 4a and b illustrates the scanning electron microscope (SEM) surface morphological image of the nano-composite (Ag@Ag_2_O) at different magnifications. The Ag_2_O exhibited bundles of nearly spherical nanoparticles ranged from 19 to 59 nm and this result is nearly consistent with the result from XRD patterns in Fig. 2a. The silver oxide may have formed in as a solution mixture containing potassium hydroxide and n-propanol which have high oxidation potential^30^. Also, the time spent since the beginning of the reaction, i.e. when adding silver nitrate to the oxidized mixture and until the end of the reaction is not enough to produce the silver oxide in its final form. Thus, it is an incomplete reaction that results in the precipitation of the silver nanopowders. This step entails the formation of a layer of silver oxide on the surface of the silver powder nanoparticles as a result of remaining in the oxidizing solution for a longer time^31^. Thus, it is logical to form an Ag/Ag_2_O core shell compound of a spherical nature as a result of the lattice mismatch between silver metal and silver oxide^6^. However, the aggregation is more likely to occur due to too small size as shown in Fig. 4a and b. Generally, the smaller particle size is usually more beneficial for the antibacterial activity. Because the particle size is smaller, many more particles will be easily adsorbed on the surface of the bacterial cell membrane, and then successfully attack the cell, ultimately destroying the physiological functional groups of the cell^32^.

#### Transmission electron microscopy (TEM)

TEM has been employed to characterize the size, shape, morphology and crystallinity of the synthesized Ag@Ag_2_O NCs. Figure 4c–i shows both TEM and HRTEM images of the Ag@Ag_2_O NCs where regular spherical shaped nanoparticles with size range of 40–60 nm are detected which is in good agreement with the particle size calculated from the SEM images. In Fig. 4e, core shell nanoparticle is clearly indicating the existence of Ag as core and Ag_2_O as shell shape. High-resolution TEM (HRTEM) in Fig. 4e shows the lattice fringes with a d-spacing of 2.33 Å, which matches to the (111) reflection of face-centered cubic (fcc) of Ag. The crystallinity of Ag@Ag_2_O was observed by selected area emission diffraction (SAED), which was recorded by directing the electron beam perpendicular to NCs and shown in Fig. 4i. That reveals the polycrystalline nature of the Ag@Ag_2_O. The elemental mapping of Ag and O elements shown in Fig. 4f–h shows a major distribution of Ag element with regular and minor distribution of O element. The detected multi diffraction spots in Fig. 4i may be referred to the dual lattices from both Ag and Ag_2_O nanostructures.

### Zeta potential

**Figure 5:**
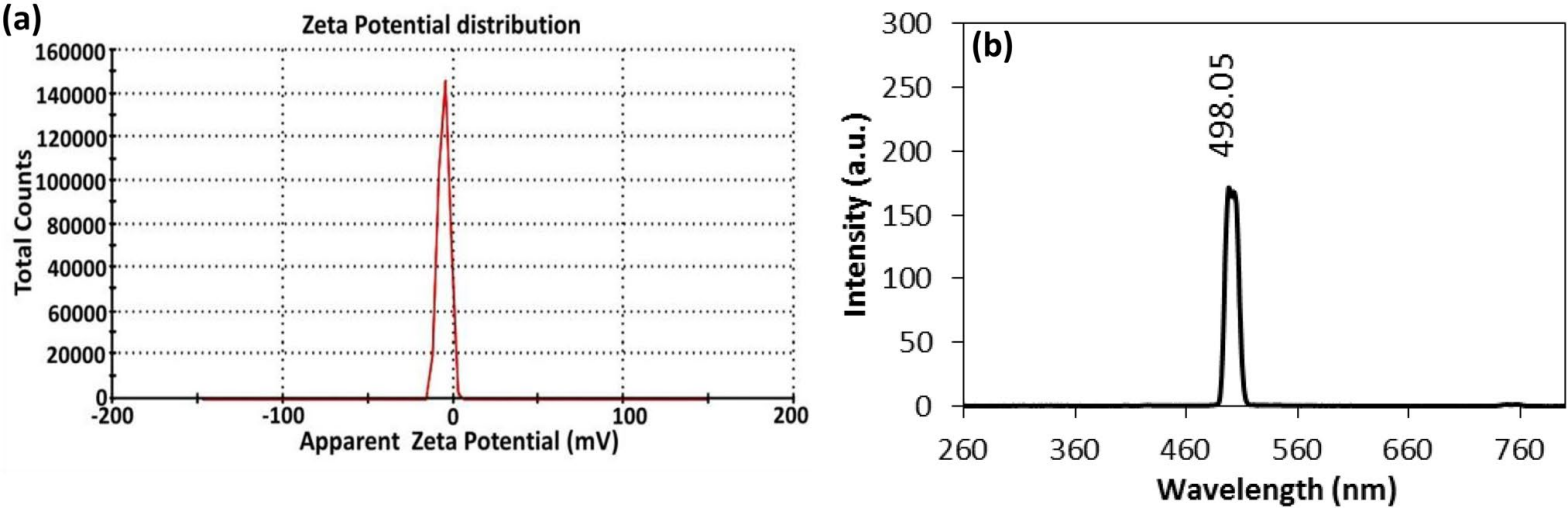
(**a**) Zeta potential and (**b**) Fluorescence emission spectrum of Ag@Ag_2_O NCs.

The surface charge of Ag@Ag_2_O core shell was determined from Zeta potential by applying voltage across a pair of electrodes at either end of a cell containing the particle dispersed. The charged particles are attracted to the oppositely charged electrode and assessing the Zeta-potential value by − 5.34 mV (Fig. 5a). The Ag@Ag_2_O NCs show slightly low surface charges which tend to form agglomerates^33^. Moreover, the low surface charges of Ag@Ag_2_O NCs reflect the urgent requirement of a capping agent to prevent such agglomeration and keep nanocomposites stable for a long time^34^. However, upon antimicrobial application and cytotoxicity evaluation, the examined NCs were freshly prepared and examined after a short time of preparation (within 48 h of preparation). Subsequently, the prepared NCs, within such time, didn’t exhibit aggregation and were still stable. Additionally, several reports^35,36^ synthesized AgNPs and other metal-NPs in the same range of zeta and also exhibited antimicrobial activity.

### Fluorescence spectra

The fluorescence emission peak of Ag@Ag_2_O NCs was detected using an excitation wavelength of 250 nm and appeared at about 498 nm in the visible range as shown in Fig. 5b. This fluorescence emission peak may be attributed to the relaxation of the electronic motion of surface plasmons^37^. The sharpening behavior in the peak may be due to the core shell structure and coverage of Ag by Ag_2_O, which prevents the nanopowder from combining with any water molecules as well as continuing the oxidation process^38^.

### The chemical mechanism

Based on the preceding experimental data, it is worth mentioning to explain the chemical mechanism of the nanocomposite (Ag@Ag_2_O) formation as demonstrated in Eq. (3). The reaction of silver nitrate with potassium hydroxide produces silver hydroxide via the following mechanism^24^:

3 $$\mathrm{AgN}{\text{O}}_{3}+\mathrm{KOH }\to \mathrm{AgOH}+{K}^{+} +\mathrm{N}{\text{O}}_{{3}^{-}}$$

The intermediate AgOH is thermodynamically unstable, and the Ag_2_O is formed phase through many steps, as shown in the following Eqs. (4)–(7). Briefly, a part of AgOH may be reacting with the n-propanol, which acts as a wetting agent that decreases the recombination rate and the generation of silver propanoate (Ag-O_2_CCH_2_CH_3_), as shown in Eq. (4), which is inferred from FTIR spectra as a sharp peak at 1655 cm^−1^ and 3400 cm^−1^ as shown in Fig. 2c ^39^. Meanwhile, Ag-O_2_CCH_2_CH_3_is reacted with the hydroxyl group of KOH producing silver ions (Ag^+^) in a continuous oxidation process [Eq. (5)]. The silver ion reacts with water and n-propanol in an alkaline medium via the presence of OH^-^ group to produce silver element (core); as shown in [Eq. (6a)]. Additionally, some of the silver ions re-interact with water and n-propanol for producing silver hydroxide as in [Eq. (6b)]. Therefore, the unstable silver hydroxide product (AgOH) is reduced to silver oxide (Ag_2_O shell) as shown in [Eq. (7)].

4 $$2\mathrm{AgOH}+{2\mathrm{H}}^{+}({\mathrm{OCH}}_{2}{\mathrm{CH}}_{2}{\mathrm{CH}}_{3}{)}^{-}\to 2{\mathrm{Ag}}^{+}-({\mathrm{O}}_{2}{\mathrm{CCH}}_{2}{\mathrm{CH}}_{3}{)}^{-}+{2\mathrm{H}}_{2}\mathrm{O}+ {2\mathrm{H}}_{2}\uparrow$$

5 $$2{\mathrm{Ag}}^{+}-({\mathrm{O}}_{2}{\mathrm{CCH}}_{2}{\mathrm{CH}}_{3}{)}^{-}+2{\mathrm{OH}}^{-}+ {2\mathrm{H}}_{2}\mathrm{O }\to 2{\mathrm{Ag}}^{+}+2{\mathrm{HOCH}}_{2}{\mathrm{CH}}_{2}{\mathrm{CH}}_{3}+2{\mathrm{OH}}^{-}$$

6a $$2{\mathrm{Ag}}^{+}+2{\mathrm{HOCH}}_{2}{\mathrm{CH}}_{2}{\mathrm{CH}}_{3 }+2{\mathrm{OH}}^{-}+ {\mathrm{H}}_{2}\mathrm{O }\to {2({\mathrm{O}}_{2}{\mathrm{CCH}}_{2}{\mathrm{CH}}_{3})}^{-}+2{\mathrm{H}}_{2}\mathrm{O}+\mathrm{Ag}$$

OR

6b $$2{\mathrm{Ag}}^{+}+2{\mathrm{HOCH}}_{2}{\mathrm{CH}}_{2}{\mathrm{CH}}_{3}+2{\mathrm{OH}}^{-}+ {\mathrm{H}}_{2}\mathrm{O }\to 2\mathrm{Ag}-\mathrm{OH}+{\mathrm{H}}_{2}\mathrm{O}+2{\mathrm{HOCH}}_{2}{\mathrm{CH}}_{2}{\mathrm{CH}}_{3}$$

7 $$2\mathrm{AgOH }\to {\mathrm{Ag}}_{2}\mathrm{O}+ {\mathrm{H}}_{2}\mathrm{O}$$

### Antimicrobial efficiency of Ag@/Ag_2_O NCs against planktonic pathogens

**Figure 6:**
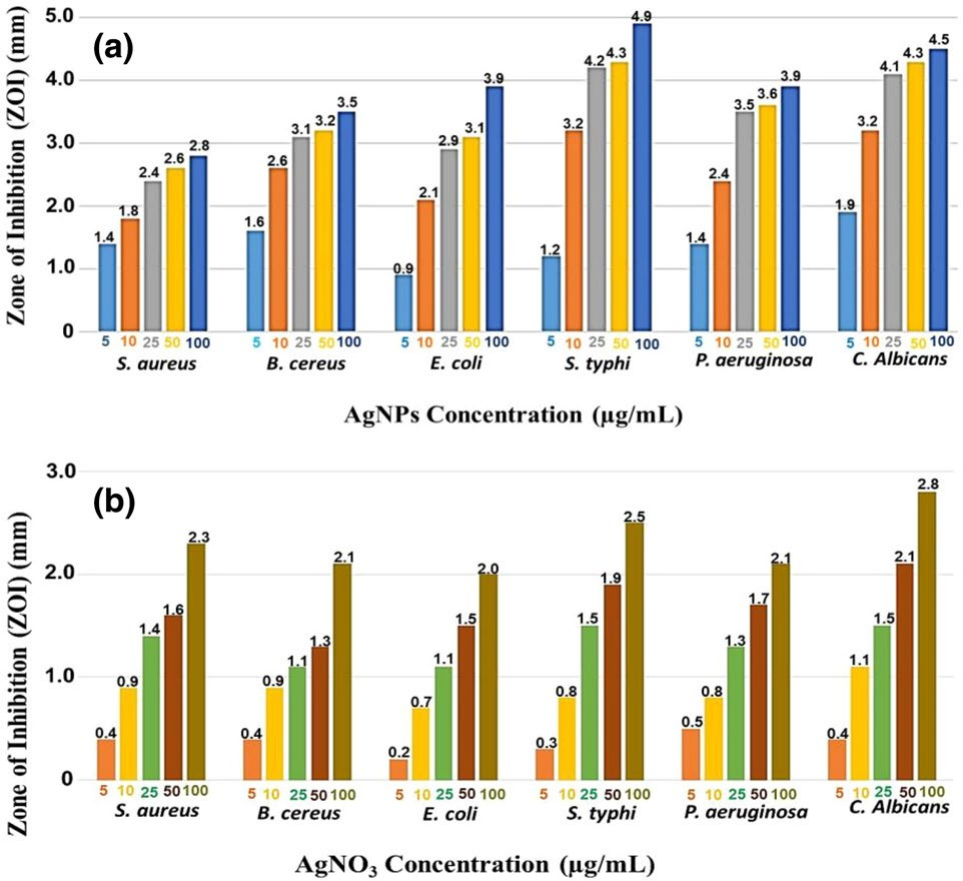
Antimicrobial activity of different concentrations of Ag@Ag_2_O NCs (**a**) and AgNO_3_ (**b**) against some prokaryotic and eukaryotic pathogens.

**Figure 7:**
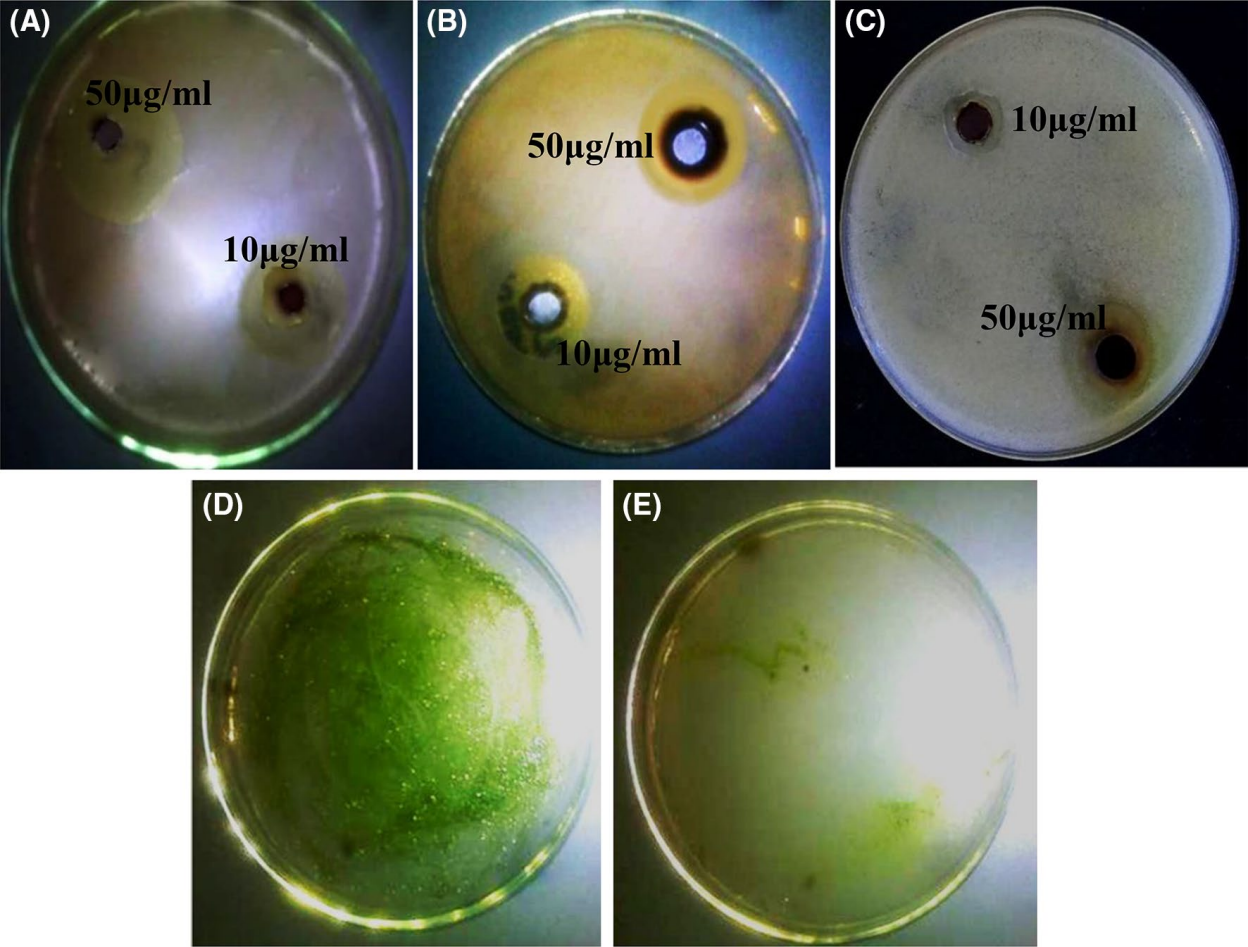
Antimicrobial activity of different concentrations of Ag@Ag_2_O NCs against *E. coli* (**A**), *B. cereus* (**B**) and *C. albicans* (**C**) as representative models of pathogenic Gram-negative bacteria, Gram-positive bacteria and Fungi, respectively. (**D**) *C. vulgaris* control before treatment with Ag@Ag_2_O NCs and (**E**) *C. vulgaris* after treatment with 50 μg/ml of Ag@Ag_2_O NCs.

Considering the health problems associated with microbial contamination, it is vital to find out effective antimicrobial agents that are able to control their outbreak. Thus, the current study is concerned with the antimicrobial activity of Ag@Ag_2_O NCs against some prokaryotic and eukaryotic pathogens. The sensitivity of the examined pathogens to different concentrations of Ag@Ag_2_O NCs is shown through agar diffusion assay. Figure 6a and b illustrates the comparative results of antimicrobial activities of the Ag@Ag_2_O NCs and their precursor. Also, it demonstrated a dose-dependent manner in which the antimicrobial activity of different concentrations of Ag@Ag_2_O NCs against *E. coli*, *B. cereus* and *C. albicans* as representative models of pathogenic Gram-negative bacteria, Gram-positive bacteria and Fungi, as well as *C. vulgaris* control before treatment with Ag@Ag_2_O NCs and *C.vulgaris* after treatment with 50 μg/mL of Ag@Ag_2_O NCs are shown in Fig. 7A–E respectively. Generally, Ag NPs displayed considerable effectiveness indicated by halo zones which exceeded 1 mm, where any antimicrobial agent was evaluated as “good” atan inhibition zone greater than 1 mm^40^. For all the examined pathogens, inhibition halos were directly proportional to the concentration of AgNPs. In addition, Gram-positive strains seemed to be more resistant than Gram-negative strains. That could be attributed to the lipophilicity of Ag NPs according to different cell wall polarity and compositional variations^41^.

As revealed by Pazos-Ortiz et al.^42^ the thickness of the cell wall increases the resistance of bacteria to the exposed NPs. The thick peptidoglycan layer of the Gram-positive bacteria's wall, which is composed of teicoic acids and lipoteicoic acids, restricts the diffusion of NPs. Moreover, the tolerance response of each microbe depends on its metabolic properties. However, the cell wall of the Gram-negative bacteria is composed of thinner peptidoglycan layer together with lipoprotein and lipopolysaccharide, which together represent 25% of its mass. It is noteworthy to mention that the nosocomial infections and enteric fever are associated with *P. aeruginosa*, *E. coli* and *S. typhi*, respectively. Therefore, their inhibition is a pivotal issue. In agreement with our results^42,43^ reported low reduction in *S. aureus* count (CFU/mL) and also halo zone in comparison to Gram-negative bacteria upon treatment by Ag@Ag_2_O NPCs. Besides, Ag@Ag_2_O NPCs biosynthesized by aqueous leaf extract of *Eupatorium odoratum* (EO) exhibited antagonistic performance coincident with the obtained results of current study^41^. In the same sense, D’Lima et al.^6^ reported that Ag/Ag_2_O hybrid nanoparticles showed a considerable zone of inhibition against *P. aeruginosa*; declaring the enhancement of antibacterial activity upon combination with carbenicillin. In contrast, other studies reported higher susceptibility of Gram-positive bacteria for NPs treatment than Gram-negative one^11,44^. Remarkably, a considerable halo of mycostasis was noticed against *C. albicans*. Despite the oligodynamic nature of silver ions, which is due to their higher activity at minute concentrations, a potent antifungal efficiency of 50 μg of Ag@Ag_2_ONCs exhibited upon comparing with its precursor (Fig. 6); implying effectiveness in the treatment of COVID-19 post infections. Such fungal infections appeared recently in the second wave in India, in particular in patients who were put on mechanical ventilation in intensive—care units. The fungicidal property of Ag@Ag_2_ONCs could be assigned to the damage of the glycoprotein-glucan-chitin cross-linkage of fungi cell wall followed by sever alterations in cellular biochemistry^11,45^. In addition, it has been suggested that Ag nanoparticles interact with the proteins of the plasma membrane, which is responsible for keeping trans-membrane electrochemical potential gradient such as H^+^ ATPase protein. Such interaction leads to alterations of normal protein conformations and malfunctioning by blocking the regulation of H^+^ transport across the membrane, which ultimately hindering growth, restraining respiration and ending with death^46–48^. In coincidence with our results, Mallmann et al.^49^ highlighted similar results with inhibitory influence of Ag@Ag_2_O NCs against several species of *Candida.* Otherwise, Elemike et al.^41^ demonstrated the dominant biocidal effectiveness ofAg@Ag_2_O NCs in bacteria than fungi.

### Evaluation of the as-prepared Ag@Ag_2_ONCs against biofilm formation, biofilm disintegration and algal growth

Biofilms are multicellular sessile microbial communities embedded in a self-produced extracellular polymeric matrix (EPS) (e.g. DNA, proteins and polysaccharides) and attached toa living or inert substratum or interface. Actually, the viscoelastic nature of the EPS represents a serious concern, especially in water pipes, water purification systems and also in medical devices. Where, the biofilms have the capability to withstand different stress factors by the virtue of such feature. Hence, nanotechnology invasion has provided a significant tool to eradicate such problem at both environmental and medical levels^50^. The inhibitory effect of different concentrations of as-synthesized Ag@Ag_2_ONCs and their precursor salt on biofilm formation/ disintegration of both Gram-positive and Gram-negative bacteria was illustrated in Table 1. As noticed, *P. aeruginosa* biofilm was less susceptible for both treatments and under formation/ disintegration conditions, in comparison to *S. aureus* biofilm. As revealed by Hoseini -Alfatemi et al.^51^, *P. aeruginosa* and *S. aureus* biofilms were inhibited by 10 and 1 mg/mL of AgNPs, respectively; which makes our study characteristic. Where, 100 µg/mL suppressed (98.7% and 87.5%) and (93.1 and 74.8%) of *S. aureus* and *P. aeruginosa* biofilm synthesis and disintegration, respectively. Interestingly, Gram‐negative biofilms were comparatively more resistant to antibiofilm treatments than Gram‐positive as reported in several studies^42,51,52^. Generally, Ag@Ag_2_O NCs exhibited antibiofilm activity via several routes including, destruction of initial planktonic phase, damage of aggregated/sessile phase, disruption of EPS matrix, increasing of hydrophobicity of EPS and inhibition of quorum sensing system^53^.

**Table 1:** Biocide activity of Ag@Ag_2_O NCson bio-film formation, biofilm disintegration and algal growth.

Antimicrobial agent	Concentration µg/mL	Biofilm formation		Biofilm disintegration		*C. vulgaris*
		*S. aureus*	*P. aeruginosa*	*S. aureus*	*P. aeruginosa*	
		Inhibition %				
Ag@Ag_2_O NCs	5	50.7 ± 2.4	49.3 ± 1.7	37.3 ± 1.0	27.5 ± 1.9	38.46 ± 3.3
	10	62.4 ± 1.3	58.6 ± 1.4	44.6 ± 2.3	35.8 ± 2.1	52.74 ± 2.7
	25	75.3 ± 1.1	67.3 ± 2.8	60.2 ± 2.1	53.5 ± 1.0	76.37 ± 1.9
	50	89.2 ± 2.7	81.4 ± 1.1	74.7 ± 1.4	67.8 ± 1.1	83.64 ± 2.1
	100	98.7 ± 2.5	93.1 ± 1.1	87.5 ± 0.9	74.8 ± 1.5	98.45 ± 2.0
AgNO_3_	5	10.5 ± 1.8	7.2 ± 0.9	8.1 ± 1.8	5.5 ± 0.4	7.4 ± 1.0
	10	19.1 ± 2.3	16.7 ± 2.5	13.7 ± 0.6	10.1 ± 0.4	16.3 ± 0.8
	25	33.8 ± 2.7	28.6 ± 3.1	21.2 ± 0.9	18.3 ± 1.5	21.5 ± 1.9
	50	45.1 ± 1.0	41.4 ± 1.5	33.6 ± 0.7	29.4 ± 1.7	33.4 ± 2.5
	100	58.3 ± 1.3	49.7 ± 1.4	43.5 ± 2.4	39.7 ± 2.1	41.5 ± 2.1

What is more, the inhibitory effect of Ag@Ag_2_ONCs against algal growth of *C. vulgaris *was studied*. C. vulgaris* is involved among other algal genera which are responsible for various environmental issues such as eutrophication and biofouling, especially in the availability of high concentrations of contaminants and in association with direct sunlight^53^. As illustrated in Table 1, Ag@Ag_2_O NCs exhibited a drastic algicidal effect on the proliferation and viability of algae with 98.4% growth inhibition. Severe damage of chloroplasts could be proposed due to yellowish to pale green color of algal growth in the presence of Ag@Ag_2_ONCs. Meanwhile, the control culture (without Ag@Ag_2_ONCs) appeared green and flourished during 7 daysof incubation as shown in Fig. 7D and E. Disintegration of algal cell organelles, thylakoid disorder and plasmolysis are common features associated with the destructive effect of Ag@Ag_2_ONCs on algal cell as stated by Duong et al.^54^. Therefore, the employment of Ag@Ag_2_ONCs in restriction the algal blooms could result in constraining of their environmentally adverse influence.

As general observations, Ag@Ag_2_ONCs exhibited greater inhibitory activity than its precursor against all examined microbial forms. That could be assigned to the small size of nanoparticles and in relation to surface area. As pointed out by^55^, the antagonistic activity of NPs derived from their penetration ability which depends on sizes that are less than 100 nm. In addition, the biocide activity of Ag@Ag_2_ONCs uplifted linearly with increasing in Ag@Ag_2_O NCs concentration, which implies dose-dependent manner. However, NPs type, concentration, size, aggregation state, surface charge, synthesis conditions and tested microbe consider being governing parameters influencing of the effective doses^51^.

Broadly, several strategies could be ascribed for NPs to display their toxicity against different microbial forms. The first strategy begins from puncturing and perforating the first protective barrier of the cell, which is cell wall, by interacting with its anionic components such as neuraminic acid, N-acetylmuramic acid, and sialic acid. However, as long as the NPs are smaller than 80 nm, their passage to cell membrane and later inside the cell is facile; causing phospholipid peroxidation, polysaccharides depolymerization and subsequently membrane detachment and integrity destruction^10,56^. At this stage, cell permeability increases followed by intracellular components leakage and proton motive force dissipation. Once NPs occupies intracellularly, more destructive features were exerted concerning metabolism and biochemical activities^10^. AgNPs showed higher affinity for binding with thiol group of amino acids; forming extra –S–S– bonds. By such way, deformation of protein configuration occurs, leading to proteins denaturation and ribosomes inactivation^56,57^. Further, NPs bind with nucleic acids such genomic and plasmid DNA; causing blockage of DNA replication and repair processes. With continuous release of Ag^+^ ions and their oxide from Ag@Ag_2_O NCs, set of reactions (e.g., Fenton and Haber–Weiss reactions) are continuously and intensively generating Reactive Oxygen Species (ROS) such as hydroxyl radicals (OH^−^), superoxide radicals (O_2_^−^) and singlet oxygen (^1^O_2_). Under such oxidative stress, massive damage to the cell takes place and eventually lead to cell death. Tee et al.^58^ and Pazos-Ortiz et al.^42^ referred to the complexity of the mechanisms by which NPs exhibit their antagonistic influence. Figure 1b represents schematic illustration on the destructive effect of Ag@Ag_2_O NCs against different microbial forms.

### Cytotoxicity assessment

**Figure 8:**
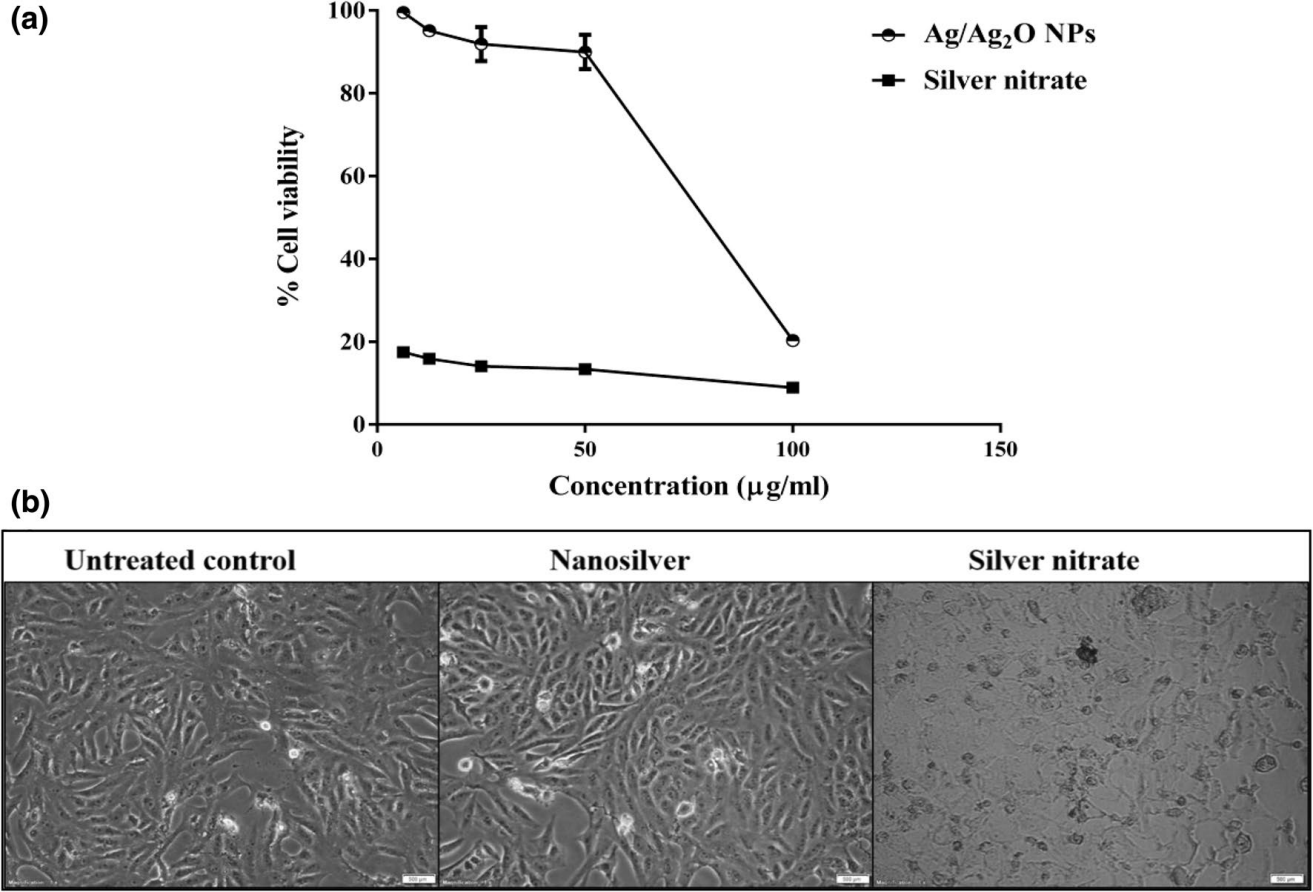
Cytotoxicity of Ag/Ag_2_O NCs compared to silver nitrate on Vero cell line. (**a**) Cell viability % after 72 h incubation with serial concentrations of Ag/Ag_2_O NCs and silver nitrate using MTT assay with (**b**) morphology of Vero cells after 72 h incubation with 25 µg/ml of Ag/Ag_2_O NCs and silver nitrate (magnification × 200).

After 72 h of incubation of the Ag/Ag_2_O NPs and silver nitrate precursor with normal renal epithelial Vero cells, it was found that their estimated safe doses on cell viability were 13.43 ± 1.63 µg/mL and 0.075 ± 0.001 µg/mL, respectively. This indicates that Ag/Ag_2_O NPs ismore safe than silver nitrate source. However, at 100 µg/mL of Ag/Ag_2_O NPs or silver nitrate caused death in Vero cells by 79.69% and 91.09%, respectively, as it is shown in Fig. 8a. Moreover, severe collapse in the normal spindle shape of silver nitrate-treated cells, at 25 µg/mL, confirmed its cytotoxicity in comparison to the normal morphology of Ag/Ag_2_O NPs-treated cells and untreated control healthy cells (Fig. 8b)^14^. The lower cytotoxicity of the prepared NPs, at < 13 µg/mL, may be related to their particle size (≥ 40 nm), negatively particle charge and high agglomeration potential (Fig. 4a,b) which results in increasing their size thus decreasing their cellular uptake and diminishes ROS generation^59^.

In support of this issue, Liu et al.^60^ found that Ag NPs with size of 55 nm generated less ROS than 15 nm Ag NPs. Moreover, silver NPs’ tendency to agglomeration increases in culture medium^61^. Besides, based on the previous finding, corona formation which is mediated by adsorption of fetal bovine serum (FBS), from culture medium, on silver NPs, mainly limits their cytotoxicity via reducing their cellular uptake^59,62^. All these factors contribute to minimize the cytotoxicity effect of Ag/Ag_2_O NCs on normal cells. This higher safety of Ag/Ag_2_O NPs on human normal cells (Fig. 1c) lends credibility to their biomedical applications compared to bulk silver nitrate.

## Conclusion

Due to the globally identified antibiotic resistance among clinical pathogens, novel antimicrobial materials are needed to circumvent drug resistance. In this study, we have demonstrated a facile chemical method to fabricate Ag@Ag_2_O core–shell nanocomposites and their antibacterial, antifungal and antibiofilm activities against a wide range of microbial pathogens were examined. Structural, morphological and optical properties were studied using different techniques. XRD, Raman, FTIR, XPS and SAED indicated the formation of a hybrid NC structure with a crystalline nature. SEM and HRTEM showed the evidence of Ag@Ag_2_O with a spherical core–shell structure and its particle size ranging from 19 to 60 nm.

Furthermore, the antagonistic properties of Ag@Ag_2_O core–shell and its precursor AgNO_3_ were compared in the range of 5–100 μg/mL. The image data declared the sensitivity order of pathogens versus examined Ag@Ag_2_O as follows: *S. typhi ˃ P. aeruginosa ˃ E. coli ˃ B. cereus ˃ S. aureus*. Besides, a noticeable antifungal potency of Ag@Ag_2_O was observed at 50 μg/mL. Additionally, its antibiofilm activity and disintegration capability were increased with elevation of concentration. Generally, a dose-dependent behavior could describe the inhibition of examined pathogens by Ag@Ag_2_O. Eventually, the cytotoxicity of the NC was analyzed by Vero cells and its effective safe concentration value was estimated to be about 36.31 ± 1.53 µg/ml. The promising structural features and biocidal activity of Ag@Ag_2_O opens up employment in various technological sectors.

## Data Availability

All data generated or analyzed during this study are included in this article.
